# THE EFFECT OF MINDFULNESS-ORIENTED RECOVERY ENHANCEMENT INTERVENTION ON POSTSTROKE COGNITIVE IMPAIRMENT

**DOI:** 10.1093/geroni/igae098.1015

**Published:** 2024-12-31

**Authors:** Lijing Chen, Jing Chu, Juan Li

**Affiliations:** Huashan Hospital Fudan University, Shanghai, Shanghai, China (People’s Republic); Naval Medical University, Yangpu, Shanghai, China (People’s Republic); Huashan Hospital Fudan University, Shanghai, Shanghai, China (People’s Republic)

## Abstract

**Background:**

About one-third of stroke patients are affected by cognitive impairment, and there is evidence that Mindfulness-based interventions (MBIs) can improve cognitive function.

**Objectives:**

This study aimed to examine the effects of cognitive intervention based on Mindfulness-Oriented Recovery Enhancement (MORE) on cognitive function in patients with Post-Stroke Cognitive Impairment (PSCI).

**Methods:**

A non-randomized controlled trial was conducted. A total of 44 patients with PSCI were enrolled, with 24 in the intervention group and 20 in the control group. The control group received routine rehabilitation therapy (RT), and the intervention group received 45-60 minutes of cognitive intervention based on MORE twice a week for four weeks plus RT.

**Results:**

In the intervention group, 21 participants reached the standard of active participation. In intervention group, the mean change of global cognitive function (Montreal Cognitive Assessment, MoCA) after intervention compared with before intervention was 2.33 (95%CI: 1.73 – 2.93, p<0.001), while in the control group, the mean change of MoCA was 1.25 (95%CI: 0.77 – 1.72, p<0.001). Using a per- protocol analysis, the score of MoCA in the intervention group improved significantly compared to that in control group. The intervention group also showed significant improvement in attention, depressive symptoms, mindfulness, self-efficacy, resilience, and quality of life compared to the control group (p < 0.05, respectively).

**Conclusions:**

The cognitive intervention based on MORE is feasible and effective in improving the cognitive function of patients with PSCI, as well as relieving depressive symptoms and quality of life.

